# News and Coming Events

**Published:** 1907-03-30

**Authors:** 


### NEWS AND COMING EVENTS.

THE second international congress of Physical Therapeutics will be held in Rome in October next, under the presidency of Professor Bacellé.

Two performances of *Twelfth Night* will be given by the Merrie Andrews Dramatic Society on April 23 and 24 at the West Theatre, Albert Hall, in aid of the Mount Vernon Consumption Hospital at Hampstead and Northwood.

THE annual meeting of the Society for the Study of Inebriety will be held at 11 Chandos Street, Cavendish Square, W., on Tuesday, April 9, at 4 P.M. A short address will be delivered by the President, after which a discussion on "Alcoholism and the Poor Law" will be opened by Dr. F. S. Toogood.

THE funds of the Afsalgunj Hospital, Hyderabad, have recently received a most welcome addition. This charitable institution is entirely worked and financed by the Nizam's Government, and has consistently done good work. An endowment of 50,000 rupees has now been presented to the hospital by Brigade-Surgeon John Law, M.D. (retired), who was acting Residency Surgeon in Hyderabad some thirty years ago, and who took a keen interest in the development of the hospital.

THE annual British Homœopathic Congress will be held at Harrogate on Thursday, September 19, under the presidency of Dr. Wolston, of Edinburgh. The *British Homœopathic Review*, which is the official organ of the Homœopathic Association, is starting its new series, the first number of which contains much interesting and instructive reading. The *Review* has secured the collaboration of several eminent foreign homœopathists, among them being Drs. Tessier, Wouters, and Gisevius, and will in future pay special attention to the progress of homœopathy in America and on the Continent.

THE Medical Golf Tournament will take place on Thursday, May 30, and application has been made to the Burnham Beeches Golf Club for permission to play off the event on their links. To secure the continuity from year to year of this popular fixture, and to promote the interests of golf among practitioners, there has been founded a Medical Golfing Society, with an annual subscription of 4s., the payment of this sum including entrance to the tournament. Any person on the medical or dental registers can join by sending 4s. to the Hon. Treasurer, Medical Golfing Society, 1 New Cavendish Mansions, New Cavendish Street, London, W.

AT a meeting of the Central Committee and prominent subscribers of the Medical College Fund, held at Lucknow on December 5 last for the purpose of considering the plans of the Medical College Hospital, prepared by Sir Swinton Jacob in consultation with the Hon. Colonel Murray, Inspector-General of Civil Hospitals, it was decided that the hospital should be a double-storied building and not a single-storied building. The site is Shamina, overlooking the Victoria Park. The hospital will accommodate 200 beds, and one of the features of the plan is the attached series of cottage hospitals, twelve in number, for paying patients. The plan of the college proper and the other buildings is still under consideration.

THE annual general meeting of the Netherlands Nursing Association will be held at Utrecht on May 29 next.

THE Metropolitan Convalescent Institution, 32 Sackville Street, Piccadilly, has received a special grant of £500 from the Goldsmiths' Company.

MESSRS. JAMES BUCHANAN AND COMPANY, LIMITED, the well-known firm of whisky distillers, have been granted the imperial warrant of appointment for the supply of Scotch whisky to the imperial household of Japan.

THE annual report of the Bristol General Hospital draws attention to the urgent needs of this institution. The Committee appeals for £12,000 to pay for two important additions to the hospital buildings, the new isolation wing, and the additional wing of the Nurses' Home.

PLANS for the erection of a new cottage hospital for Dunoon have been approved by the local Dean of Guild Court. The new hospital is estimated to cost between £3,000 and £4,000, and will be built on a specially selected site off Sandbank Road, to the north of the recreation ground, facing the town. It will be two stories high, and will afford accommodation for twelve patients.

ALDERMAN SIR WALTER VAUGHAN MORGAN will preside at the festival dinner in aid of the funds of the Royal Hospital for Incurables, Putney Heath, at the Whitehall Rooms, on May 16. The cost of the maintenance of the institution is £35,000 a year. With this money the hospital maintains over 200 incurable patients at Putney, and, still further, sends throughout Great Britain over 700 pensions of £20 a year to incurable men and women who are in possession of a home, but who have no means of support.

H.R.H. PRINCE CHRISTIAN, the Danish Minister, the Portuguese Minister, the Greek Minister, the Swedish Minister, the Archbishop of Canterbury, the Bishop of London, the Archbishop of Westminster, the Chief Rabbi will support H.R.H. the Prince of Wales at a dinner at which he has graciously consented to preside, to be held at the Hôtel Cecil on May 14, in aid of the National Sanatorium for Workers suffering from Tuberculosis. Mr. Alfred de Rothschild has kindly consented to lend his band for the occasion.

THE list of speakers at the annual meeting of the London Temperance Hospital included Lord Alverstone, Lady Biddulph, and Sir John W. Benn, M.P. This hospital was founded in 1873 for the treatment of disease without the administration of alcohol. Alcohol is, however, prescribed and administered when it is thought necessary by the medical staff. The new Out-patients' Hall is nearly completed at a cost of £4,000, of which £400 has yet to be subscribed. There should be no difficulty in raising many thousands of pounds of revenue for a temperance hospital from small subscribers if an administrative organisation were created for the purpose. The revenue the annual subscriptions raise represents little more than a tenth of the total expenditure.

